# Food-Derived Bioactives Can Protect the Anti-Inflammatory Activity of Cortisol with Antioxidant-Dependent and -Independent Mechanisms

**DOI:** 10.3390/ijms17020239

**Published:** 2016-02-15

**Authors:** Erik J. B. Ruijters, Guido R. M. M. Haenen, Mathijs Willemsen, Antje R. Weseler, Aalt Bast

**Affiliations:** Department of Pharmacology and Toxicology, Faculty of Health, Medicine and Health Sciences, Maastricht University, Maastricht 3600 MD, The Netherlands; erik_ruijters@hotmail.com (E.J.B.R.); mathijs.willemsen@mumc.nl (M.W.); a.weseler@maastrichtuniversity.nl (A.R.W.); a.bast@maastrichtuniversity.nl (A.B.)

**Keywords:** cortisol, flavonoids, glucocorticoid, inflammation, antioxidant

## Abstract

In chronic inflammatory diseases the anti-inflammatory effect of glucocorticoids (GCs) is often decreased, leading to GC resistance. Inflammation is related with increased levels of reactive oxygen species (ROS), leading to oxidative stress which is thought to contribute to the development of GC resistance. Plant-derived compounds such as flavonoids are known for their ability to protect against ROS. In this exploratory study we screened a broad range of food-derived bioactives for their antioxidant and anti-inflammatory effects in order to investigate whether their antioxidant effects are associated with the ability to preserve the anti-inflammatory effects of cortisol. The anti-inflammatory potency of the tested compounds was assessed by measuring the oxidative stress–induced GC resistance in human macrophage-like cells. Cells were pre-treated with H_2_O_2_ (800 µM) with and without bioactives and then exposed to lipopolysaccharides (LPS) (10 ng/mL) and cortisol (100 nM). The level of inflammation was deducted from the concentration of interleukin-8 (IL-8) in the medium. Intracellular oxidative stress was measured using the fluorescent probe 2′,7′-dichlorofluorescein (DCFH). We found that most of the dietary bioactives display antioxidant and anti-inflammatory action through the protection of the cortisol response. All compounds, except for quercetin, revealing antioxidant activity also protect the cortisol response. This indicates that the antioxidant activity of compounds plays an important role in the protection of the GC response. However, next to the antioxidant activity of the bioactives, other mechanisms also seem to be involved in this protective, anti-inflammatory effect.

## 1. Introduction

Our diet contains a wide variety of antioxidants and other bioactives which can help to preserve health. These health benefits have been extensively documented in literature and various modes of action have been proposed [1,2,3,4]. It is increasingly realized that a bioactive has a multitude of subtle effects via diverse cellular and molecular targets that become integrated in an overall, physiologically relevant response [5]. One of the best-documented activities of flavonoids, an important group of bioactives, is their ability to protect against reactive oxygen species (ROS) [6,7,8,9].

ROS are generated during the reduction of oxygen and can be derived from sources as diverse as mitochondria, activated inflammatory cells, drugs and cigarette smoke. ROS comprise two groups of molecules, namely (i) free radicals with relatively short biological half-lives, such as superoxide anion (O_2_^−^) and hydroxyl radical (OH); and (ii) nonradicals, such as hydrogen peroxide (H_2_O_2_) and hypochlorous acid (HOCl), which are relatively less reactive and have a longer half-life than free radicals [10]. ROS are noxious molecules and can damage virtually any cellular components. Beside the oxidative stress–mediated direct damage by ROS, the induction of inflammation by ROS has also been implicated in the etiology of numerous diseases [10,11,12].

Inflammation is a natural process to combat infections and help in wound healing. During this process immune cells are attracted to the area of inflammation and various cytokines (e.g., tumor necrosis factor α (TNF-α) and chemokines (e.g., interleukin-8 (IL-8)) are produced. It is a feed-forward process that can spin out of control, leading to a chronic inflammatory response. Inflammatory mediators activate the nuclear factor kappa-light-chain-enhancer of activated B cells (NF-ĸB), initiating cytokine production and leukocyte activation. This in turn increases ROS production, resulting in antioxidant depletion and oxidative stress, which further stimulates NF-ĸB [13,14].

Glucocorticoids (GCs) are the most effective anti-inflammatory drugs available for the treatment of many chronic inflammatory and immune diseases, including inflammatory bowel disease (IBD) and chronic obstructive pulmonary disease (COPD). The anti-inflammatory effect of GCs is often decreased in these diseases, leading to GC resistance and a state of chronic inflammation [15,16,17]. These chronic inflammatory diseases are also associated with an increased level of oxidative stress, which is thought to contribute to the development of GC resistance [18]. This again illustrates how closely inflammation and oxidative stress are intertwined.

In previous research, we showed that the cocoa flavanol (−)-epicatechin (EC) was able to preserve the anti-inflammatory effect of the endogenous GC cortisol [19] and the synthetic GC dexamethasone in oxidant-exposed human macrophages [20]. These results revealed that the antioxidant activity of EC prevents the development of GC resistance in macrophage-like cells. In the present study we examined a wide array of bioactives, with a focus on flavonoids and some of their metabolites, for their antioxidant and anti-inflammatory effect, *i.e.*, the preservation of the anti-inflammatory effect of cortisol, in oxidant-exposed cells and correlated this to their antioxidant activity. Our data will enhance the understanding of the structural relation between the antioxidant activity of dietary bioactives and their potency to protect the cortisol response.

## 2. Results

### 2.1. Antioxidant Effect of Bioactives and Protection against GC Resistance

**Figure 1:**
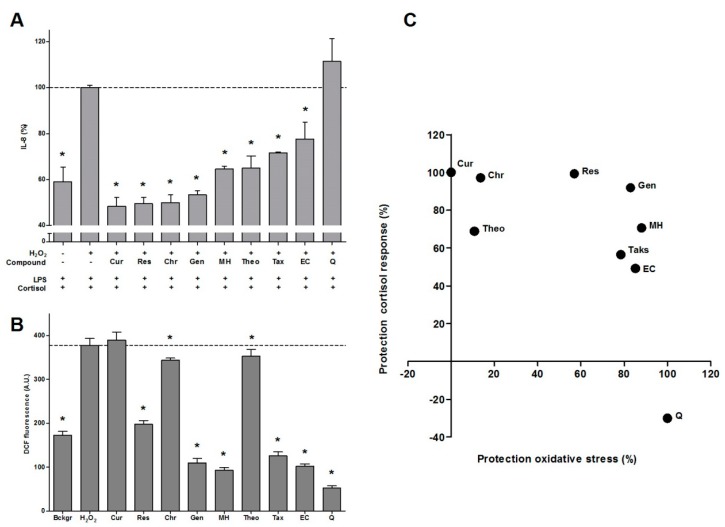
Effect of bioactives on intracellular oxidative stress and glucocorticoid resistance arranged in accordance to their ability to restore the anti-inflammatory effect of cortisol. (**A**) IL-8 levels (%) in medium after cells were pre-incubated with H_2_O_2_ (800 µM) ± bioactives (10 µM) for 1 h. and subsequently exposed to LPS (10 ng/mL) and cortisol (100 nM) for 16 h (*N* = 6, mean ± SEM); (**B**) Intracellular oxidative stress levels. Differentiated monocytes were incubated with DCFH for 45 min and then exposed to H_2_O_2_ (800 µM) ± bioactives (10 µM) and fluorescence was recorded for 1 h (*N* = 4, mean ± SD, * *p* < 0.05 Dunnett’s); (**C**) Correlation of the ability to reduce intracellular oxidative stress and to protect the cortisol response. Dotted lines represent maximal IL-8 production or fluorescence because no bioactives were added during H_2_O_2_ incubation.

A wide array of dietary compounds (10 µM) was tested for their protective effect against oxidative stress and GC resistance in differentiated U937 cells (Table 1). The bioactives efficiently protected the cells against oxidative stress–induced GC resistance (mean ± S.D.): Cur (100% ± 10%) ≥ Res (99% ± 14%) ≥ Chr (97% ± 9%) ≥ Gen (92% ± 13%) ≥ MH (71% ± 16%) ≥ Theo (69% ± 13%) ≥ Tax (56% ± 11%) ≥ EC (49% ± 44%) (Figure 1). Res, Gen, MH, Tax, Q, and EC also protected against oxidative stress and reduced DCF fluorescence even below background levels. The data depicted in Figure 1 indicate that the tested compounds can be divided into two groups: one consisting of Cur, Res, Chr and Gen, and another composed of MH, Theo, Tax and EC.

The bioactives that displayed antioxidant activity also protected the GC response of cortisol, except for Q. Although Q reduced intracellular stress even below background level, this flavonol did not reduce the LPS-induced IL-8 production in the macrophage-like cells. This lack of protection indicates that more mechanisms are involved in the development and prevention of GC resistance. This is also illustrated by the compounds Theo, Chr and Cur which show the opposite: little to no antioxidant activity but efficient protection of the GC response by 69%, 97% and 100%, respectively.

### 2.2. Antioxidant Effect of Flavonoid Metabolites and Protection against GC Resistance

**Figure 2:**
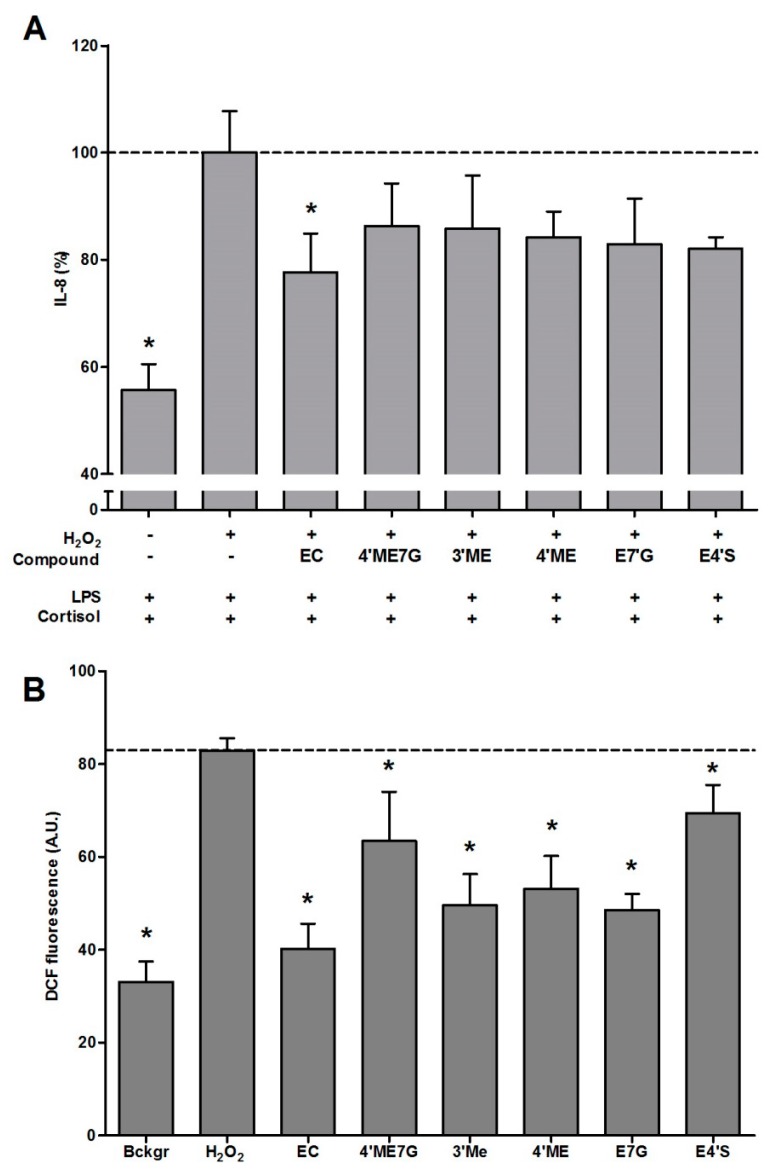
Effect of the flavanol EC and several metabolites on intracellular oxidative stress and glucocorticoid resistance. (**A**) Cells were pre-incubated with H_2_O_2_ (800 µM) ± flavanols (10 µM) for 1 h and subsequently exposed to LPS (10 ng/mL) and cortisol (100 nM) for 16 h. IL-8 levels (%) were measured in the cell medium (*N* = 6, mean ± SEM); (**B**) Intracellular oxidative stress. Differentiated monocytes were incubated with DCFH for 45 min, exposed to H_2_O_2_ (800 µM) ± flavanols and fluorescence was measured for 1 h (*N* = 4, mean ± SD, * *p* < 0.05 Dunnett’s). The dotted line represents IL-8 production or fluorescence of cells pre-incubated with H_2_O_2_ (800 µM) without the bioactives, and subsequently exposed to LPS (10 ng/mL) and cortisol (100 nM).

**Figure 3:**
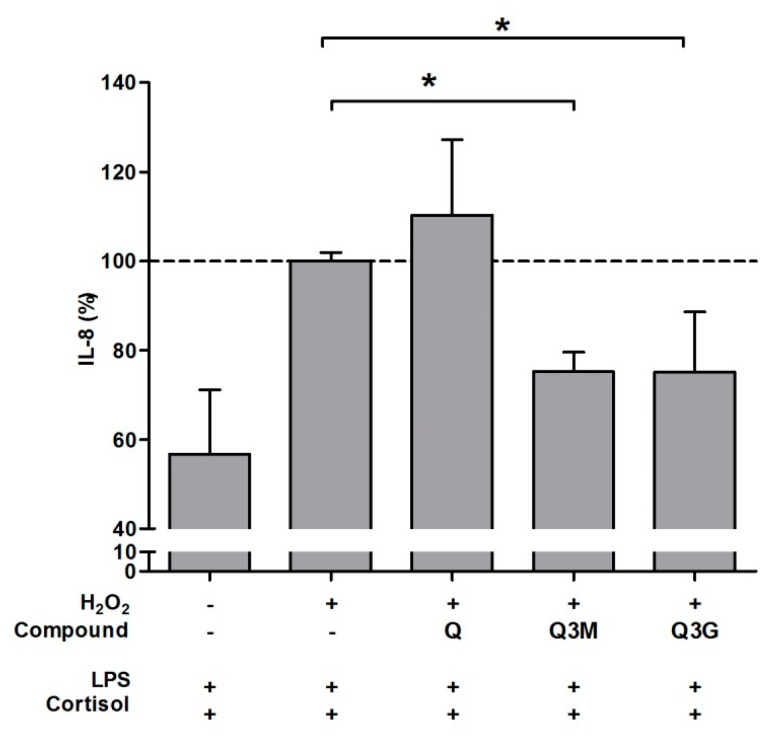
Effect of quercetin (Q) metabolites on cortisol response. Cells were pre-incubated with H_2_O_2_ (800 µM) ± Q metabolites (10 µM) for 1 h and subsequently exposed to LPS (10 ng/mL) and cortisol (100 nM) for 16 h and IL-8 levels (%) measured in the cell medium (*N* = 6, mean ± SD, * *p* < 0.05 Mann-Whitney *U* test). The dotted line represents IL-8 production of cells pre-incubated with H_2_O_2_ (800 µM) without the bioactives, and subsequently exposed to LPS (10 ng/mL) and cortisol (100 nM).

Several metabolites were investigated for their antioxidant and GC-protective effect (Figure 2 and Figure 3). Metabolism, *i.e.*, methylation, glucuronidation or sulfation, especially at the 3′and 4′ carbon, results in a reduced antioxidant capacity. This was illustrated by the increased DCF fluorescence compared to the parent compound EC. However, the protective effect of the metabolites against GC resistance was similar to the protection by EC. Although Q appeared to enhance the IL-8 production in our cell model, its 3-*O*-methyl and 3-*O*-glucuronide metabolite reduced IL-8 release by 57% and 55%, respectively. Both Q metabolites thus provide protection against oxidative stress–induced GC resistance.

**Table 1:** Antioxidant effect and protection of the cortisol response by bioactives (10 µM).

Compound	Abbr.	Antioxidant Effect (%)	Protection Cortisol Response (%)
**Flavanol**			
(−)-epicatechin	EC	86 ± 11	49 ± 44
3′-*O*-methyl(-)-epicatechin	3′ME	67 ± 13	36 ± 53
4′-*O*-methyl(-)-epicatechin	4′ME	56 ± 14	39 ± 31
(−)-epicatechin-7-*O*-β-d-glucuronide	E7G	69 ± 7	42 ± 46
4′-*O*-methyl(−)-epicatechin-7-*O*-β-d-glucuronide	4′ME7G	39 ± 21	35 ± 46
(−)-epicatechin-4′-*O*-sulfate	E4′S	27 ± 12	41 ± 12
**Flavanonol**			
(±)-Taxifolin	Tax	78 ± 3	56 ± 11
**Flavone**			
Chrysin	Chr	14 ± 2	97 ± 9
**Flavonol**			
7-mono-*O*-(β-hydroxyethyl)-rutoside	MH	88 ± 2	71 ± 16
Quercetin	Q	100 ± 2	−30 ± 47
3-*O*-methyl-quercetin	Q3M	-	57 ± 6
Quercetin-3-*O*-glucuronide	Q3G	-	55 ± 24
**Isoflavone**			
Genistein	Gen	83 ± 3	92 ± 13
**Curcuminoid**			
Curcumin	Cur	−4 ± 6	100 ± 10
**Stilbenoid**			
Resveratrol	Res	57 ± 3	99 ± 14
**Methylxanthine**			
Theophylline	Theo	11 ± 4	69 ± 13

## 3. Discussion

Bioactives from dietary sources have numerous health benefits which are achieved via a multitude of subtle effects. Best understood is the antioxidant activity, which could also contribute to the myriad of effects such as their anti-inflammatory potential. In this exploratory study we determined the antioxidant effect and the capacity to protect the GC response of a wide array of food-derived compounds. Correlation of these data revealed to what extent these effects are interrelated for the structurally different compounds. Additionally, metabolites of the flavanol EC and the flavonol Q were tested, since flavonoids are extensively metabolized in the gastro-intestinal tract and upon entering the circulation [21,22].

We found that most of the food-derived bioactives displayed antioxidant and anti-inflammatory activity through the protection of the cortisol response. All compounds, except for Q, showing antioxidant activity also protected the cortisol response. This indicates that the antioxidant activity of compounds plays an important role in the protection of the GC response. Since H_2_O_2_-induced oxidative stress impaired the anti-inflammatory effect of cortisol, the antioxidant activity of EC could be directly linked to the protection of the GC response in our previous study [19]. Compounds such as EC, MH, Gen and Tax display prominent antioxidant effects and provide protection for the GC response. The only exception seems to be Q, which showed a high antioxidant activity but no protection of the GC response. This finding was unexpected since other studies have reported anti-inflammatory effects of Q [23,24]. The absence of the anti-inflammatory activity in our test system may be explained by the fact that, in an environment high in oxidants, Q may be converted into thiol-reactive quinone products as a result of the radical scavenging. These quinones can react with protein thiols, causing reduction or loss of their function [25,26].

The bioactives Cur, Chr and Theo display little to no antioxidant activity but protect the GC response, indicating that the protection against oxidative stress is not the only way to maintain the anti-inflammatory effect of GCs. This is also corroborated by the results of the metabolites of EC. Although EC metabolites have a reduced antioxidant capacity, as illustrated by an increase in intracellular oxidative stress compared to the parent compound EC, the cortisol response is partially protected by these metabolites. Apparently, more molecular mechanisms could be involved in the protection of the GC response.

Several investigated bioactives, including their metabolites, are known for these pleiotropic cellular and molecular effects. Previous studies have shown that curcumin and theophylline improve the GC response, presumably by restoring histone deacetylase 2 (HDAC2) activity [27,28]. Another mechanism that increases the anti-inflammatory effects of glucocorticoids is the inhibition of the enzyme phosphodiesterase 4 (PDE4) [29]. Flavonoids have been reported to exert beneficial effects on the cardiovascular system by the inhibition of PDE, which elevates cyclic adenosine monophosphate (cAMP) levels and leads to an activation of the cAMP-dependent protein kinase A (PKA) [30]. As a consequence, microvascular leakage is diminished, and trafficking and release of cytokines and chemokines from inflammatory cells is inhibited [31]. Another possible mode of action is the activation of peroxisome proliferator activated receptor α (PPARα). PPARs are transcription factors belonging to the nuclear hormone receptor family and play a central role in the regulation of lipid, lipoprotein and glucose metabolism. PPARs have also been identified as important regulators of inflammatory gene expression [32,33]. Activated PPAR can inhibit the pro-inflammatory transcription factor NF-ĸB, and it has been demonstrated that various isoflavones are potent PPARα and PPARγ agonists [34]. By activating PPARα, these isoflavones can reduce the NF-ĸB-mediated pro-inflammatory gene expression and attenuate the inflammatory response. To what extent all these different mechanisms are involved in the action of the investigated compounds needs to be addressed in further studies.

In summary, we show that various food-derived bioactives have antioxidant and anti-inflammatory activity by protecting the anti-inflammatory effect of cortisol in human macrophages exposed to oxidative stress. Next to the antioxidant activity of the bioactives, other mechanisms also seem to be involved in this protective, anti-inflammatory effect. This opens new options for the treatment of inflammation and chronic inflammatory diseases, by boosting the anti-inflammatory effect of the endogenous GC cortisol.

The absence of a direct correlation between the antioxidant effect and the capacity to protect the cortisol response suggests that other molecular mechanisms are also involved in the activity of the investigated, nutritionally relevant bioactives. The observation that a wide variety of plant-derived food compounds, including metabolites, are able to preserve the endogenous GC response opens new possibilities for dietary approaches specifically targeted for patients with chronic inflammatory diseases.

## 4. Materials and Methods

### 4.1. Chemicals

Phorbol 12-myristate 13-acetate (PMA), lipopolysaccharides (LPS) 2′,7′-dichlorofluorescein-diacetate (DCFH-DA), quercetin, chrysin, genistein, (±)-taxifolin, curcumin, resveratrol and theophylline were purchased from Sigma (St. Louis, MO, USA). Roswell Park Memorial Institute 1640 (RPMI 1640) medium, fetal calf serum (FCS), phosphate buffered saline (PBS), penicillin, and streptomycin were obtained from Gibco (Life Technologies, Carlsbad, CA, USA). The (−)-Epicatechin (EC), quercetin-3-*O*-glucuronide (Q3G), quercetin-3-*O*-methyl (Q3M) were purchased from Extrasynthese (Genay, France). 7-mono-*O*-(β-hydroxyethyl)-rutoside (MH) was kindly provided by Novartis Consumer Health (Nyon, Switzerland). The flavanol metabolites 3′-*O*-methyl-EC (3′ME), 4′-*O*-methyl-EC (4′ME), 4′-*O*-methyl-EC-7-*O*-β-d-glucuronide (4′ME7G), EC-7-*O*-β-d-glucuronide (E7G), and EC-4′-*O*-sulfate (E4′S) were provided by Mars Incorporated (Hackettstown, NJ, USA). Stock solutions of 2–10 mM of compounds were prepared in 50% or 100% (*v*/*v*) ethanol and 50 μL aliquots were stored at −80 °C until use. The dilutions of each flavanol were prepared immediately prior to experimental use.

### 4.2. Cell Culture

The human monocyte cell line U937 (LGC standards, Teddington, UK) was cultured in RPMI 1640 medium supplemented with 10% FCS and 50 U/mL penicillin and 50 µg/mL streptomycin in a humidified atmosphere with 5% CO_2_ at 37 °C. Before exposure, cells were differentiated to macrophage-like cells using 50 ng/mL PMA treatment for 4 h. After incubation, cells were plated in a 24-well plate (4 × 10^5^ cells/well) and allowed to differentiate for 48 h. Medium was changed to 1% FCS for a further overnight incubation. Cells were washed with PBS and all exposures were performed in medium without FCS. After each experiment, cell amount was determined using the sulforhodamine B (SRB) assay as previously described [19].

### 4.3. Intracellular Oxidative Stress

The fluorescent probe of 2′,7′-dichlorofluorescein-diacetate (DCFH-DA) was used to quantify intracellular oxidative stress in differentiated monocytes. In the presence of ROS, the non-fluorescent DCFH is oxidized to the highly fluorescent dichlorofluorescein (DCF). The intensity of DCF fluorescence corresponds to the level of intracellular ROS formation. Cells were differentiated in a black/clear bottom 96-well plate and grown for 72 h. Fifty µM DCFH-DA was added to the cells and incubated for 30 min. at 37 °C, 5% CO_2_. After washing, cells were exposed to 100 µL serum-free medium containing H_2_O_2_ ± bioactive and DCF fluorescence was measured with excitation and emission wavelengths of 485 and 535 nm, respectively, at 37 °C over a period of 60 min.

### 4.4. Determination of Inflammation and GC Resistance

Inflammation and GC resistance were determined in a previously developed assay [19]. Oxidative stress was induced by incubating differentiated monocytes with H_2_O_2_ (800 µM) for 60 min. Bioactives (10 µM) were added during the incubation with H_2_O_2_. Cells were washed with PBS and incubated with LPS (10 ng/mL) ± cortisol (100 nM) for 16 h. The interleukin-8 (IL-8) concentrations in the medium were measured using a commercially available ELISA kit (Sanquin, Amsterdam, The Netherlands) according to manufacturer’s protocol.

### 4.5. Statistics

Data are expressed as mean ± SD or mean ± SEM. Statistical comparisons were performed by using a Mann-Whitney *U* test or ANOVA following Dunnett’s multiple comparison test with Prism v5 (GraphPad Software, San Diego, CA, USA). A two-tailed *p*-value <0.05 was considered statistically significant.

## 5. Conclusions

The study shows that several food-derived bioactives can protect the anti-inflammatory activity of cortisol.
